# Dr. Robert C. Williams

**Published:** 1860-09

**Authors:** 


DR. ROBERT C. WILLIAMS.—Dublin has recently met with a serious loss by the decease of this accomplished physician and surgeon, in the fifty-second year of his age. In 1836, Dr. Williams was appointed Professor of Materia Medica in the Royal College of Surgeons in Ireland, and two years later became one of the Surgeons of the City of Dublin Hospital. As a lecturer he was forcible and highly instructive, and by his kind and gentle manners he endeared himself to his pupils, patients, and the profession at large. Dr. Williams was an able writer, as is well shown by his many and varied contributions to the *British and Foreign Medico-Chirurgical Review*. He had been President of the Royal College of Surgeons of Ireland.

